# Timing of Insulin Glargine 300 U/ML: Does It Really Matter in Terms of Efficacy and Safety at Insulin Initiation?

**DOI:** 10.7759/cureus.80050

**Published:** 2025-03-04

**Authors:** Debmalya Sanyal, Asis Mitra

**Affiliations:** 1 School of Medicine and Public Health, The Newcastle University, Newcastle, AUS; 2 Endocrinology, Kali Prasad Chowdhury Medical College and Hospital, Kolkata, IND; 3 Endocrinology, Narayana Health, Rabindranath Tagore International Institute of Cardiac Sciences, Kolkata, Kolkata, IND

**Keywords:** daytime administration, efficacy, insulin glargine u300, nighttime administration, noctural hypoglycaemic, safety

## Abstract

Background: The timing of insulin administration, particularly for long-acting insulins like insulin glargine 300 U/mL (Gla-300), plays a critical role in diabetes management. The choice between daytime and nighttime administration of Gla-300 is a nuanced decision that should be tailored to each patient’s unique circumstances. This study aims to compare the efficacy and safety profile of insulin initiation with Gla-300 when administered during the day versus at night in adult type 2 diabetes mellitus (T2DM) patients.

Methods: A retrospective observational study included records of adult T2DM patients initiated on Gla-300 with follow-up data of three months. Cases with complete medical records, including hemoglobin A1C (HbA1C) levels, fasting plasma glucose (FPG), post-prandial plasma glucose (PPPG), and incidence of hypoglycemic events, were included. Data pertaining to glycaemic control and safety, were extracted and categorized into two groups based on the timing of Gla-300 administration. Records were evaluated over three months and results were statistically analysed.

Results: A total of 169 patients, matched for baseline characteristics, showed a significant reduction in glycaemic indices from initiation to the third month. However, intergroup difference was non-significant suggesting that the timing of administration (morning vs. night) does not significantly impact the effectiveness of Gla-300 in lowering the glycemic indices. Incidences of overall hypoglycemic events were numerically lesser in those with morning dosing of Gla-300 at 13.09% as compared to those with night dosing of Gla-300 at 21.17%. Nocturnal hypoglycaemia was numerically lesser in those with morning dosing of Gla-300 at 3.57% as compared to those with night dosing of Gla-300 at 7.05% observed. However, these intergroup differences were insignificant (p> 0.05).

Conclusion: Timing of Gla-300 initiation, morning compared to night dosing, does not significantly impact the effectiveness in lowering the glycemic indices. Incidences of overall and nocturnal hypoglycemic events were numerically lower with morning dosing of Gla-300 compared to night dosing of Gla-300, though it was not statistically significant. An individualized approach to timing and selection of insulin therapy is essential for optimizing glycemic control and minimizing hypoglycemia risk.

## Introduction

Insulin glargine 300 U/mL (Gla-300) is a novel glargine formulation that shows slower and more prolonged absorption following subcutaneous administration in comparison to insulin glargine 100 U/mL. This long-acting basal insulin analog, used to manage blood glucose levels in individuals with diabetes mellitus, provides consistent and prolonged blood glucose control, making it a crucial component of diabetes management. Gla-300 has a higher concentration (300 units/mL) compared to its predecessor, insulin glargine U100, providing more prolonged and stable insulin release, lasting up to 36 hours [1]. It has a delayed onset of action, typically beginning within six hours of injection, and it reaches a steady-state concentration after a few days of consistent dosing. Unlike some other basal insulins, it has no pronounced peak, which helps maintain steady insulin levels. The flat pharmacodynamic profile of Gla-300 further minimizes fluctuations in blood glucose levels, reducing the risk of both hyperglycemia and hypoglycemia rendering steady insulin levels. Moreover, the formulation of Gla-300 allows for a more gradual absorption into the bloodstream, providing a consistent basal insulin supply [2].

The timing of insulin administration, particularly for long-acting insulins like Gla-300, plays a critical role in diabetes management. Moreover, the hypoglycaemia risk is higher at insulin initiation and may hinder compliance and continuation of insulin therapy. Both daytime and nighttime administration have their own benefits and drawbacks, which must be weighed carefully to optimize patient outcomes. The choice between daytime and nighttime administration of Gla-300 is a nuanced decision that should be tailored to each patient’s unique circumstances and lifestyle [3]. Both timing options offer distinct advantages and challenges. Daytime administration may reduce the risk of nocturnal hypoglycemia and align better with patients' routines, while nighttime administration provides stable overnight insulin levels and helps control fasting glucose [3]. Studies have shown that maintaining a consistent timing of Gla-300 administration, whether in the morning or evening, results in stable glycemic control. The EDITION clinical trials, specifically EDITION 1, 2, and 3, demonstrated that patients who administered Gla-300 at the same time each day achieved similar reductions in hemoglobin A1C (HbA1C) levels compared to glargine 100 U/mL, with less risk of hypoglycemia [4].

Whilst there have been studies studying the consistent timings of Gla-300 administration, there exists a dearth of real-world evidence exploring the effect of dosing timing on the efficacy and safety profile of the patients. This study thus aims to compare the efficacy and safety profile of Gla-300 when administered during the day versus at night at insulin initiation.

## Materials and methods

Study design and ethics

A retrospective observational study included records of adult type 2 diabetes mellitus (T2DM) patients initiated on Gla-300 with follow-up data of three months. The study procedures were approved by the Human Research Ethics Committee of Allergy Asthma Research Centre vide approval no AARC/54. The study was conducted according to the Declaration of Helsinki.

Study population

Adult (more than 18 years) T2DM patients initiated on Gla-300 with at least three months follow-up data pertaining to glycaemic control and safety were extracted. They were categorized into two groups based on the timing of Gla-300 administration, morning or night time. The timing of insulin was as per patient’s choice and convenience. Cases with complete medical records, including HbA1c levels, fasting plasm glucose (FPG), post-prandial plasma glucose (PPPG) and incidence of hypoglycemic events, were included. Records were evaluated over three months and statistically analysed. There were no restrictions with regards to the use of non-hypoglycaemic anti-hyperglycaemic agents (AHA) like metformin, sodium glucose co-transporter 2 (SGLT2) inhibitors, dipeptidyl peptidase-4 (DPP-4) inhibitors, or glucagon-like peptide-1 (GLP-1) agonists. 

Patient records having incomplete data or lost to follow-up cases or those with discontinuation of Gla-300 were excluded. Those on any other bolus insulin and oral hypoglycaemic agents like sulphonylureas or glinides were also excluded, which could affect the incidence of hypoglycaemic events between the groups. 

Data collection

Data pertaining to glycaemic efficacy including FPG, PPBG and HbA1c and safety including documented incidence of hypoglycaemia, overall and nocturnal, were extracted from the existing patient database at the facility and assessed. Nocturnal hypoglycaemia was defined according to the timing between 12 am and 5.59 am. 

The extracted data was categorized into two groups based on the timing of Gla-300 administration: Daytime administration - Group A; Nighttime administration - Group B.

Patient records were evaluated for FBS, PPBS, HbA1c, and hypoglycemic episodes over three months. 

Statistical analysis

The data collected was checked for completeness and then statistically analyzed. Descriptive analysis for continuous variables was summarized by mean and standard deviation, whereas the categorical variables were summarized by frequency and percentage. Where possible, demographic and categorical data were analyzed with parametric or non-parametric tests as applicable, to explore how variables differ with respect to the timing of Gla-300 administration. A p-value of less than 0.05 was considered significant. Normality test using the Shapiro Wilk test showed that the measures were normally distributed, and hence parametric approach was undertaken. All statistical analysis for various measures was performed using various statistical software packages like SPSS version 21.0 (IBM Corp., Armonk, NY, USA) and Microsoft Excel (Redmond, WA, USA).

## Results

Patient characteristics

The study included a total of 169 patients. Mean age of the study population was assessed as 59.88 years with a standard deviation of 9.43 years. Males formed 55.62% (n=94) of the study population, with the gender ratio being 1.25:1 (Male: Female). The common comorbidities observed were hypertension, hyperlipidaemia. Mean duration of diabetes was 12.8 years, and mean BMI was observed as 28.25 kg/m2. For both groups, variables like age, gender, comorbidities, duration of diabetes and BMI were matched, and no significant difference was observed between the groups (Table 1).

**Table 1:** Baseline Characteristics BMI - Body Mass Index. Continuous variables (e.g. Age, Average duration of diabetes, BMI) represented as mean and standard deviation (SD), test statistics calculated as t value. Categorical variables (e.g. Gender, Comorbidities) represented as number and frequency, test statistics calculated as chi square value. P value less than 0.05 considered significant.

Variables	Group A (N=84)	Group B (N=85)	Test Statistics	Intergroup Differences (p value)
Age (in years) [Mean ±SD]	60.17 ± 9.65	59.65 ± 9.11	0.36	0.719
Gender (Male/Female) [n]	50/34	44/41	1.03	0.31
Comorbidities [n(%)]				
Hypertension	60 (71.42%)	58 (69.04%)	0.204	0.651
Hyperlipidaemia	64 (76.19%)	66 (78.57%)	0.05	0.822
Average duration of diabetes (in years)	12.5 ± 6.3	13.1 ± 6.0	0.634	0.526
BMI [Mean ±SD]	28.4 ± 4.5	28.1 ± 4.7	0.424	0.672

Main findings

Efficacy Measures

**Figure 1:**
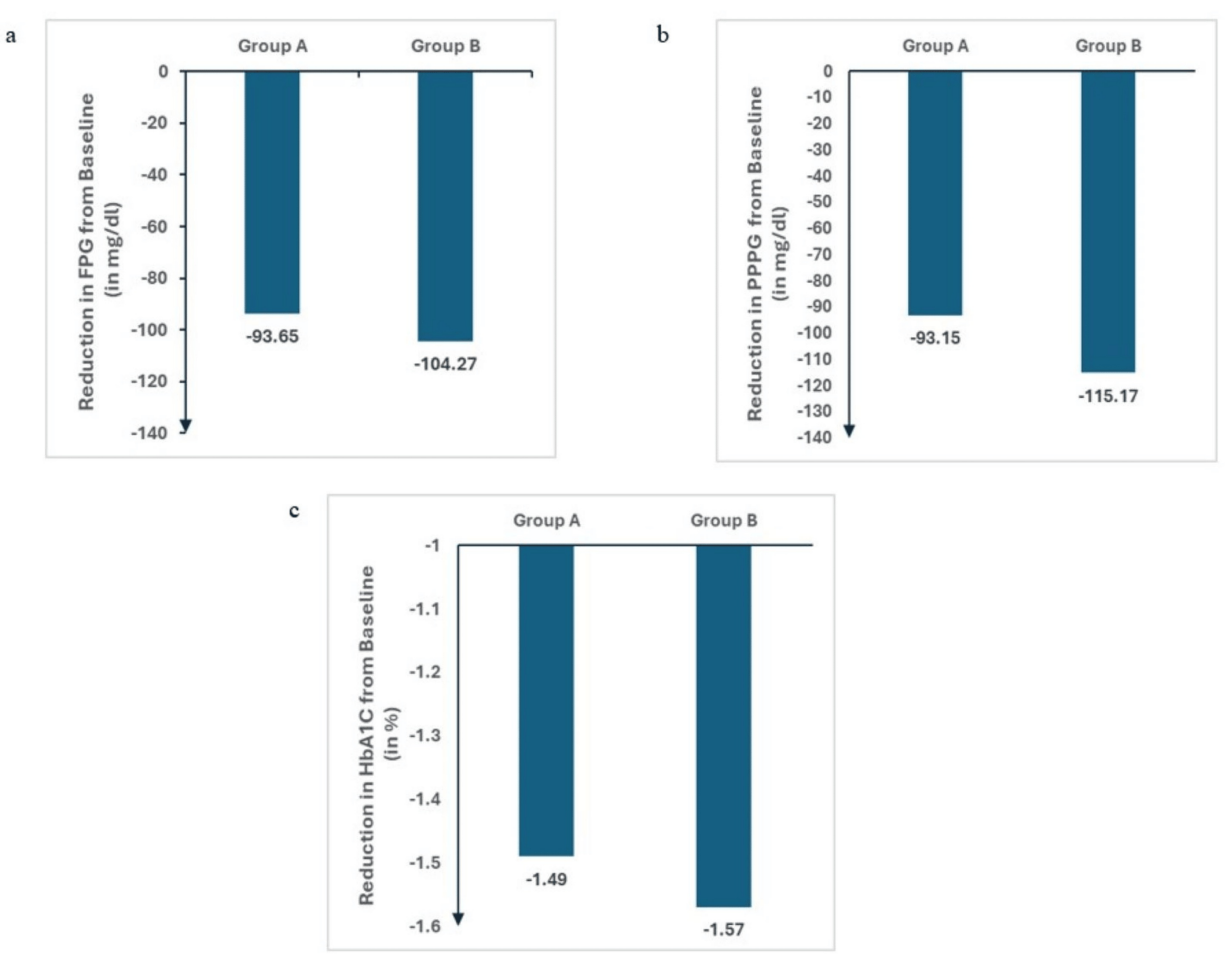
Change in (a) FPG (b) PPPG (c) HbA1C from baseline for both groups. Group values represented as mean change of parameter from baseline. FPG - Fasting Plasma Glucose; PPPG - Post Prandial Plasma Glucose; HbA1C - Glycosylated Haemoglobin.

Efficacy measures included glycaemic indices like FPG, PPPG and HbA1C. Both groups showed a significant reduction in FPG, PPPG and HbA1C from baseline to the third month. The lack of significant intergroup difference at baseline and the comparable reductions in FPG, PPPG and HbA1C suggest that the timing of administration (morning vs. night) does not significantly impact the effectiveness of Gla-300 in lowering the glycemic indices like FPG, PPPG and HbA1C (Table 2, Figure 1).

**Table 2:** Efficacy Measures FPG - Fasting Plasma Glucose; PPPG - Post Prandial Plasma Glucose; HbA1C - Glycosylated Haemoglobin. Continuous variables (FPG, PPPG and HbA1C) represented as mean and standard deviation (SD), test statistics calculated as t value. P value less than 0.05 considered significant.

Variables	Time of Measure	Group A (N=84)	Group B (N=85)	T value	Intergroup Differences (p value)
FPG	Baseline [Mean ±SD]	214.30 ± 92.60	225.92 ± 91.83		
	3rd Month [Mean ±SD]	120.64 ± 33.72	121.65 ± 28.20		
	Change from Baseline [Mean ±SD]	93.65 ± 82.39	104.27 ± 86.96	0.815	0.35
PPPG	Baseline [Mean ±SD]	262.73 ± 105.84	276.81 ± 110.75		
	3rd Month [Mean ±SD]	169.57 ± 56.09	161.64 ± 53.28		
	Change from Baseline [Mean ±SD]	93.15 ± 88.02	115.17 ± 101.21	1.508	0.131
HbA1C	Baseline [Mean ±SD]	8.66 ± 1.54	8.82 ± 1.38		
	3rd Month [Mean ±SD]	7.17 ± 0.81	7.24 ± 0.92		
	Change from Baseline [Mean ±SD]	1.49 ±1.24	1.57 ± 1.11	0.442	0.336

Safety Measures

**Figure 2:**
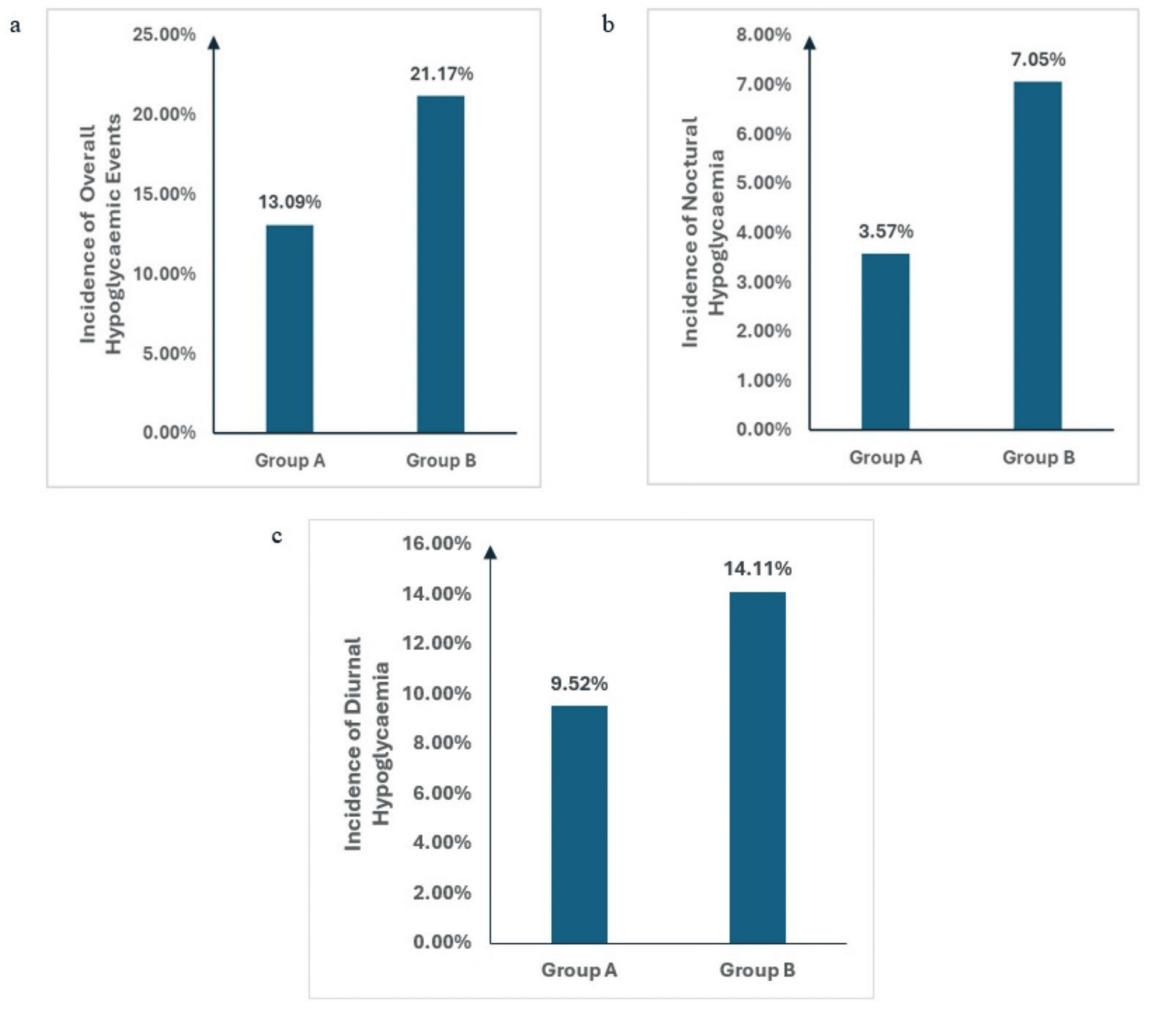
Incidence of Hypoglycaemic events - (a) Overall (b) Noctural (c) Diurnal hypoglycaemia in both groups

Incidences of overall hypoglycemic events were numerically lesser with night dosing compared to morning dosing of Gla-300. Noctural hypoglycaemia was also numerically lower with morning dosing compared to night dosing of Gla-300. However, these intergroup differences were not statistically significant (p> 0.05) (Table 3, Figure 2).

**Table 3:** Incidence of Hypoglycaemia Group values represented as frequency and percentages. P value less than 0.05 considered significant.

Events	Types	Group A (N=84)	Group B (N=85)	Chi Square Statistics	Intergroup Difference (p value)
Hypoglycaemic Events	Overall	11 (13.09%)	18 (21.17%)	1.941	0.163
	Nocturnal	3 (3.57%)	6 (7.05%)	1.019	0.312
	Diurnal	8 (9.52%)	12 (14.11%)	0.854	0.355

## Discussion

An individualized approach to timing and selection of insulin therapy is essential for optimizing glycemic control and minimizing hypoglycemia risk. Our study found that initiating insulin therapy with Gla-300, irrespective of timing of administration, morning or night dosing, had significant improvements in FPG, PPPG, and HbA1c from baseline to the third month without any significant intergroup difference. Thus similar magnitudes of improvement in glycaemic control across all measures indicate that the timing of Gla-300 administration (morning vs. night) does not significantly impact its efficacy. The present study noted numerically lower incidence of hypoglycemic events including overall, nocturnal and daytime hypoglycaemia, with morning dose compared to nighttime administration of Gla-300, though it was not statistically significant.

Focusing more on patient-centered care, understanding a patient’s daily routine is essential [5]. For those with a consistent morning routine, daytime administration may be preferable. Conversely, those with a stable nighttime routine may benefit more from nighttime administration. By administering Gla-300 during the day, the peak action period, although minimal, occurs when the patient is awake, which allows the patient to recognize and respond to hypoglycemia symptoms more effectively [3]. Also, patients can monitor their blood glucose levels more frequently during the day. Immediate corrective actions, such as adjusting carbohydrate intake or administering rapid-acting insulin, can be taken based on real-time glucose readings. For many patients, incorporating Gla-300 into their morning routine can enhance adherence [3]. Taking insulin alongside other morning medications or breakfast can help establish a consistent dosing schedule. On the hind side, increased physical activity or delayed meals during the day can heighten the risk of hypoglycemia. Patients need to be vigilant about their glucose levels and ready to make necessary adjustments. Daytime activities, including exercise and meal timing, can be unpredictable. This variability can make it challenging to maintain stable blood glucose levels [6]. On the other hand, administering Gla-300 at night can provide stable basal insulin coverage during sleep, a period when the patient is less likely to consume food or engage in physical activity. This can help maintain target fasting blood glucose levels in the morning [7]. The nighttime period often represents a more consistent and controlled environment. Meals and activities are generally less variable, making it easier to predict insulin needs and achieve stable blood glucose levels. Nighttime administration can be particularly effective in managing the dawn phenomenon, where blood glucose levels rise in the early morning hours due to hormonal changes. Providing insulin coverage during this period helps maintain better fasting glucose levels. However, there still exists a potential risk of nocturnal hypoglycemia, especially if the insulin dose is not well-calibrated. Patients may not be able to recognize or respond to hypoglycemia while asleep, which can lead to severe hypoglycemic episodes [8]. Some patients may find it challenging to adhere to a nighttime dosing schedule, particularly if they have irregular sleep patterns or forget to take their dose before bed. Inconsistent dosing can lead to suboptimal glycemic control and increase the risk of complications.

Clinical trials comparing daytime and nighttime administration of Gla-300 generally show similar efficacy in controlling HbA1c levels [9,10]. Individual risk profiles and lifestyle preferences often determine the choice of administration timing. Some studies indicate a slight reduction in nocturnal hypoglycemia with daytime administration, but overall hypoglycemia risk can be managed with careful dose titration and patient education.

The American Diabetes Association (ADA) and the European Association for the Study of Diabetes (EASD) emphasize individualized care [11]. Insulin therapy should be tailored to patient-specific factors, including lifestyle, blood glucose patterns, and hypoglycemia risk. Combining longer-acting basal analogs (such as degludec or U-300 glargine) with oral medicines may reduce the incidence of hypoglycemia relative to U-100 glargine. Moreover the hypoglycaemia risk is higher at insulin initiation and may hinder compliance and continuation of insulin therapy. The first 12 weeks of insulin initiation is generally known as the titration phase, when the risk of hypoglycaemia is higher and can lead to early discontinuation of insulin therapy. The risk of overbasalization with insulin therapy is something that clinicians need to be aware of. Clinical signals such as basal dose of more than 0.5 units/kg, significant variability, hypoglycemia (knowing or unconscious), and bedtime-morning or post-preprandial glucose differential (e.g., bedtime-morning glucose differential >50 mg/dL) may warrant assessment of overbasalization. When overbasalization is detected, a reevaluation should be conducted in order to further individualize therapy [12].

Factors such as diet, physical activity, work schedule, and personal preferences can significantly influence insulin regimens. Exercise impacts blood glucose levels, often lowering them [13]. Patients with active lifestyles may need lower insulin doses or adjustments in timing to prevent hypoglycemia during and after physical activity [13]. Shift workers or those with irregular schedules may require flexible insulin regimens. Tailoring insulin therapy to fit their unique routines can help maintain stable blood glucose levels. Some patients prefer fewer injections or specific times for insulin administration. Understanding and incorporating these preferences can improve adherence to the insulin regimen.

Monitoring blood glucose levels helps in identifying patterns and adjusting insulin doses accordingly to achieve optimal glycemic control. Regular blood glucose monitoring helps identify trends and patterns, like if a patient experiences morning hyperglycemia (the dawn phenomenon), their nighttime insulin dose may need adjustment [12]. Based on glucose monitoring data, insulin doses can be fine-tuned. Individualized targets are set based on a patient's age, comorbidities, and overall health. Younger, healthier patients might aim for stricter glycemic control, while older patients or those with multiple comorbidities may have more relaxed targets to avoid hypoglycemia. Continuous glucose monitoring (CGM) or frequent self-monitoring of blood glucose (SMBG) provides valuable data for tailoring insulin administration timing [14].

Comorbidities, age and patterns of hypoglycaemia, especially nocturnal can guide insulin timing adjustments. Patients with a history of nocturnal hypoglycemia might consider daytime administration to reduce this risk, along with use of second-generation basal insulin analogs like Gla-300, with lower hypoglycemia risk, especially nocturnal hypoglycaemia [15]. Morning administration of Ga-300 may also be considered in older adults, who are more susceptible to hypoglycemia due to multiple factors, including slower metabolism and potential cognitive decline. Comorbidities like chronic kidney disease or liver dysfunction, which increase hypoglycaemia especially nocturnal by altering insulin metabolism and neoglucogenesis, may be better suited to morning Gla-300 administration to avoid hypoglycemia [16].

The study has certain limitations, in being a retrospective short-duration study with limited sample size. Prospective, longitudinal study with a greater sample size can ascertain the findings, reinforcing the generalizability of this result. Concomitant use of AHAs like metformin, SGLT2, DPP4 inhibitors, and GLP1 agonists could have been possible confounders and a limitation of the study.

## Conclusions

Our study found that initiating with daytime or nighttime administration of Gla-300 had comparable efficacy and safety profiles. Irrespective of timing of Gla-300 administration, there were significant improvements in glycemic indices like HbA1c, FPG and PPPG, from initiation to 12 weeks without any significant intergroup difference, indicating that the timing of Gla-300 administration (morning vs. night) does not significantly impact its efficacy. Incidences of overall and nocturnal hypoglycemic events were numerically lower with morning dosing of Gla-300 compared to night dosing of Gla-300, though it was not statistically significant. This is very important for compliance and long-term continuation of insulin therapy especially during the high-risk titration period after insulin initiation. An individualized approach to timing and selection of insulin therapy, considering patient’s choice, age, comorbidity and glycaemic profile, is essential for optimizing glycemic control and minimizing hypoglycemia risk.
